# Outraged Laws of Hygiene

**Published:** 1856-01

**Authors:** D. L. M’Gugin

**Affiliations:** Prof. of Physiology and Pathology, in the Medical Department of the Iowa University


### OUTRAGED LAWS OF HYGIENE.

BY D. L. M'GUGIN, M. D.

Prof. of Physiology and Pathology, in the Medical Department of the Iowa University.

In entering upon the work of reform in the habits of the people, with the view to the establishment of rules for the preservation of health and life, and the abolishment of practices destructive of both, many deep-rooted prejudices are to be met and overcome, and many difficulties are to be encountered. These arise from different sources—prominent among which are, prejudices inculcated in the mind in early youth, when it is not only more ready to receive impressions, but which, notwithstanding they may be erroneous and false, are very difficult to efface and are surrendered reluctantly. In after life, as we journey along, the gems of truth which glow upon our pathway are seized upon with avidity, but yet they do not fix themselves upon the mind so as to produce that sense of reality and impressiveness which characterise those which have been taught in childhood as true, although they may prove false. Yet there is a lingering, lurking bias in their favor, and a regret is felt when reason and experience teach their falsity. Then, the usages and customs of society also present a formidable obstruction in the way of amendment—for as custom becomes a law, it is very difficult to abrogate those practices which have been sanctioned by it. Then again, we are compelled to encounter, all the while, new obstacles, in the shape of newly-coined abuses, in our course, which must be met and displaced, as we progress in our efforts to disseminate truth. These are often the offspring of vanity and pride, for with every change of season there is a change of fashion. Nor is this change from bad to better, but generally from bad to *worse*, as if one sin were to beget another, and each excess to be excelled by its successor. In this way we are led on, and on, and are embarrassed by the multiplicity of fresh contrivances for evil. These new additions are tolerated because fashion endorses them, and yet no one claims that their adoption is approved by reason, or justified by experience. They are tolerated because a depraved taste has suggested them, and because, too, they are adopted by some votary of fashion who is reckless and regardless of the consequences of an abuse of nature's just and unerring laws,—by some one whose conspicuity depends upon some adscititious circumstance,and not upon sound sense or ripe judgment. In this way these absurdities spring up; in this way they are multiplied, and by these means, too, health and life are jeopardized and destroyed. Conscience, and a regard for the objects and purposes of our being, are lost sight of in this race of pride and vanity, and he who raises his voice against the sacrilege, is denounced, for his antique notions and puritanical proclivities.

But fashion is a goddess whose charms are resistless, whose shrine must be worshipped, whose abuses must be tolerated, and whose suggestions must be followed. It reconciles what is unseemly, it tolerates what is ridiculous, it gives reason to absurdities, it tortures comeliness into coarseness, caricatures good taste, and shows its contempt for propriety. It is ever on a mission of mischief, always concocting some evil design, and is industrious in its contrivances to find how far it can lead its worse than blind followers to their destruction.

With such a potent adversary for mischief; with such an enemy to fight, so securely fixed and in-walled by pride, and has so strong a hold upon the minds of its followers, the effort to overthrow its power and wither the legitimate fruits of its busy hands, will be found to require effort, patience, and perseverance. Humanity demands that we expose absurdities which are active in the work of mischief; conscience spurs us on to duty, regardless of consequences, and humanity imperatively requires it at our hands.

If reason proves an inefficient weapon, we must try others. The warrior often changes his heavy artillery for infantry when the enemy is in position unfavorable for the one and favorable for the use of the other; so must we exchange reason and argument for ridicule and railery. But we must first expend reason, where we expect to find reasonable persons, reserving the other branch of our armament for those who cannot be reached or affected by other missives.

In order then that we can reason with effect, we must begin affirmatively; must exhibit in a strong light the necessity for the enjoyment of certain elements of health, so that by contrast the public mind will be able to form its own conclusions. We must therefore enforce the necessity of fresh air, exercise, and proper food. Those among whom we mingle, and for whom we labor, are not willing to take our simple declarations as truth, especially when we have such ponderous obstructions to conversion thrown up before us. We must well understand the importance of the subject ourselves, and then we can reason understandingly —convincingly. The abstract fact that fresh air is indispensable to health is known, but why it is so, and why its diminution inflicts such serious consequences, are facts not so well understood. But say to them that without pure blood the body could not be properly nourished, and without nourishment they well know that their strength would be diminished, and also that the oxygen of the lungs is necessary to qualify the blood; and that in order to procure a sufficient supply of this constituent of the air, they must enjoy it fresh and uncontaminated. The lungs do the duty of supplying this oxygen, and if their function be in any way interrupted, a normal or needed proportion cannot reach the blood, the consequence of which is that the blood is in an unhealthy condition, and debility and depression are the consequences. The heart acts feebly, the blood vessels struggle to circulate the blood, the capillary power is weakened, the skin looses its natural color, the stomach fails to digest properly, the liver is overcharged with impure blood, and fails to separate the bile from it, the kidneys become inactive, the bowels costive, the brain torpid and inactive, and the mind clouded and confused. And why all this? Simply because as the blood supplies the tissues with nutrition, it also imparts to all the organs its vitalizing influences. But upon what part of the circulating fluid do these vivifying influences depend? Upon the red particles, and these would not be properly endowed, without oxygen, which we derive from the air by the process of respiration. And this air mustbe qualified with it, in proper proportions, in order to supply it to the blood through the agency of the lungs.

But while the lungs enjoy the requisite proportion of air for respiration, and its good offices are performed through the agency of these organs, it is absolutely necessary to health that there be a free circulation in order that each structure and organ should have their supply. If there is a deficiency in one part, there is too large a supply in another, or in all the rest; hence the necessity of keeping up an equality in the circulation, not with reference simply to the good of the part in which the supply is diminished, but also that of all the other organs and structures.

Now, to illustrate this, we will enter that large saloon, well filled with guests. The votaries of fashion are there. We find beauty "unadorned" too far and too much—for the neck is bare, and the shoulders and breast are exposed. The air within is deprived of its freshness, by the great demand for its essential constituents. Not only so, but during every hour it acquires more and more of impurities by expiration from so many lungs. That inherent want of fresh air is experienced by all, and particularly by those whose thoracic muscles are thrown out of function by compression, and when the diaphragm is not permitted to descend, from tight ligatures around the abdomen below it.

This manifest and urgent want is sought to be supplied by rushing to the cold hall; and a promenade is indulged in there, until the cold air has chilled the surface of the neck and breast, and the feet are frigid for want of warm covering, but which would be *horrid* where a small foot is in fashion's programme, and where folly claims a sacrifice.

The next morning the physician finds, when about to enjoy a breakfast after a night of professional labor and duty, a summons from the anxious mother of this poor child of fashion, which compels him to hurry down a morsel unmasticated, and away upon an errand and mission of painful duty. There lies prostrated before him the interesting being so much admired the night before for the *taste* and *style* of her thin and comfortless dress, and the *graceful* manner in which it was worn. The history of her case showed that she had left that crowded room with a shawl thrown loosely over her head and shoulders, and had rushed out into the cold damp air of midnight. The overshoes had been forgotten and she tripped her way upon the cold pave to her home, where, worn out with excitement and exertion, to the last of which she was unused at home, she threw herself into bed and unavailingly sought sleep. This was denied her, for

3

there was a restlessness which was premonitory of evil near at hand. A slumber did, however, close her eyes, from which she awoke in a shivering chill. This was followed by a violent fever, hurried breathing, cough with expectoration of viscid mucous with blood, pain under the scapulæ and side of chest, soreness of intercostal muscles when pressed upon, could not fully inspire without pain, pulse quick, wiry, and frequent, respiration doubled in numbers over the normal standard, the lips are livid, and there is a dark circle around the eye, because of the imperfect æration of the blood, the skin is hot and dry, and the abdominal respiratory muscles are unusually active.

The medical reader will readily perceive the character of the mischief which was here produced. The tresses which had demanded so much care and art to curl into flowing ringlets, now lay around in confusion and neglect; the smiling and beautiful face was changed to an expression of painful anxiety, in which there was a mingling shade of gloom; the white bare arm was soon in a ligature with an unseemly gash, through which flowed the purple current, which the night before reddened her cheek, and gave animation and life to her countenance; the chest, which was of alabaster whiteness and which fashion and vanity required so recently to be displayed, was now in gashes, from the repeated application of the scarificator; and in lieu of the indigestible and irritating creams, sweet-meats and sponge cakes, upon which she had so freely regaled herself at an unseasonable hour, she was compelled to take nauseants and salines with mercurials—in fine, she who was the day before full of animation and life, was now prostrated and in a precarious condition, and that doating parent, who but yesterday looked upon her daughter with so much pride and affection, now beheld her offspring struggling with disease, and perhaps with death, The disease proceeded unchecked and uncurbed, and in a short time, nature being no longer competent to the srruggle, she sunk down into an early tomb. And yet this was pronounced *a providence!* Was it not a *suicide*, rather? There was the *cause* plainly shown, and there was the *effect* of that cause. A dose of arsenic, or a halter about that fair neck would not more surely have done their work,—more speedily, it is true, but yet not more surely nor more certainly. Providence sends, for some purpose best known to himself, epidemic visitations over which man has but little control, and yet even in many of these instances, it is gravely maintained, the cause is more or less under human control and abatement. It is, therefore, difficult to say *what is* providential, but I think in a case as the above it is not so difficult to say *what is not.*

Now in running back over the history of the above case, and scanning each event, we find all the rules for the preservation of health, in the first instance, have been set at naught. The change of dress, the exposure of those portions of the surface most needing protection, inhaling poison from a degraded atmosphere, the sudden arrestation of the circulation in the capillary vessels, from the sudden exposure to the cold in the hall, the indulgence at an unseasonable hour in indigestible materials, the flight home in the mid-hour of the night through a cold air, the person but half protected with clothing, were circumstances which common observation and experience will condemn as presumptuous and eminently capable of producing disease and death. The medical man should not only condemn it as a violation of physical laws, but should show such to be—*moral* obliquities and even criminal. This he should do *boldly*, because his relation to society, and a solemn duty, requires it at his hands. Medical men are blameable when they wink at such practices and passively permit such to transpire without entering their solemn protest against it. The fear of sacrificing favor or patronage will never deter the truly good physician from the discharge of the sacred duties required of him, but will fearlessly, and on all proper occasions, warn those who are in the habit of violating nature's laws of the consequences of such practices.

